# Genetic Improvement of Cereals and Grain Legumes

**DOI:** 10.3390/genes11111255

**Published:** 2020-10-25

**Authors:** Muhammad Amjad Nawaz, Gyuhwa Chung

**Affiliations:** 1 Laboratory of Bio-Economics and Biotechnology, Department of Bio-Economics and Food Security, School of Economics and Management, Far Eastern Federal University, 690950 Vladivostok, Russia; 2 Department of Biotechnology, Chonnam National University, Yeosu Campus, Yeosu 59626, Korea

**Keywords:** cereal breeding, climate change, genomic selection, molecular markers, yield improvement

## Abstract

The anticipated population growth by 2050 will be coupled with increased food demand. To achieve higher and sustainable food supplies in order to feed the global population by 2050, a 2.4% rise in the yield of major crops is required. The key to yield improvement is a better understanding of the genetic variation and identification of molecular markers, quantitative trait loci, genes, and pathways related to higher yields and increased tolerance to biotic and abiotic stresses. Advances in genetic technologies are enabling plant breeders and geneticists to breed crop plants with improved agronomic traits. This Special Issue is an effort to report the genetic improvements by adapting genomic techniques and genomic selection.

By 2050, nearly 2.3 billion people will be added to the current global population, making it ~9.8 billion (Figure 1a). Although it seems slower than the population growth seen in the last four decades, i.e., during this time it grew by 3.3 billion people, but adding more people means that mankind will require more food and, at the same time, a secure and sustainable food system [1]. Alongside the population increase, climate change has emerged as a significant manmade global threat to agriculture and food security (Figure 1b). The trends in rising global temperatures have far-reaching but visible effects on the agricultural sector, i.e., reduction in crop yields [2]. The projected economic growth and per capita income suggests that the market demand for food will still grow. Particularly, the demand for cereals, owing to their role in the food, feed, and fuel industries, is projected to reach ~3 billion tons by 2050 [3]. Similarly, the demand for food products responsive to higher incomes such as vegetable oils, livestock, and dairy products, will be much higher than cereals. A 70% increase in food production is required by 2050. Particularly, developing countries would need to almost double their agricultural production (Figure 1c) [4]. In 2012, the Food and Agriculture Organization reported that the projected production growth rate of the major food products, i.e., meat, sugar cane, sugar beet, oil crops (and products), and cereals, will be slow, but overall production will still increase. A 2.4% increase in the yield of major crops (maize, rice, wheat, and soybean) is required to double their global production by 2050 (Figure 1d) [5]. Based on a meta-analysis of ~1700 model simulations, it was reported that global mean crop yields are projected to decrease about 3–10% per degree of warming above historical levels [6].

The most sustainable path to achieve food security is by increasing crop yields instead of clearing more land. This is true because during the last few decades, increased crop yields have been proven to be an effective tool in meeting the rising food demands [7]. This increase was accompanied by a better ability of crop plants to cope with biotic and abiotic stresses [8]. The key to these improvements has been the better understanding of genetic variation, molecular and cellular development pathways, and exploring crops’ wild relatives for unexplored genetic potential. The traits that can significantly enhance crop plant productivity include the identification of resistance proteins that can recognize the signals from invading pathogens and resist pathogens, survival of plants under submerged conditions, better root architecture to grow in limited water conditions, increased metal as well as salt-stress tolerance, better photosynthetic efficiency with improved light harvesting and CO_2_ fixing potential, better ability to withstand heat stress, and highly efficient nutrient uptake potential including efficient symbiotic abilities to get more benefits from natural nutrient reservoirs [9]. Plants with such traits are being developed through the application of advances in genetic technologies. Advances have been made in genetic technologies, i.e., sequencing and genotyping, genomics-assisted breeding (genomic selection, genotypic platforms, training populations, and statistical models, targeted mutation breeding, and multiplex genome editing [10]), marker-assisted selection and breeding, speed breeding, phenomics, machine learning, and integration and coordinated approaches which combined the conventional breeding, genomics, and biotechnological tools (Figure 2) [11].

This Special Issue includes a broad range of modern techniques for the genetic improvement of rice, sorghum, Chinese cabbage, field mustard, common bean, and soybean. A common feature of the papers in this Special Issue is the use of genomic techniques (alone or in combination with other plant breeding or biotechnological techniques) for the identification of genes or the exploration of pathways, demonstrating the trend in research on cereals and legumes that is illustrated in Figure 2. Extracting useful information out of whole transcriptome or proteome analyses is a challenging step; however, some of the articles included in this Special Issue, i.e., Ahmed et al. [12], Zhang et al. [13], and Chen et al. [14] have demonstrated that advances in genomics are aiding in the exploration of associated pathways and key genes in studied traits.

Genomic selection (GS) is a breeding paradigm that has been successfully implemented in crop plants to address the shortcomings of phenotypic selection such as minimizing biased marker effect estimates and enables researchers to capture more variation due to small effect quantitative trait loci (QTLs) [15]. This is achieved through incorporating genome-wide marker information in a breeding value prediction model [16]. GS has played a role in supplementing population genetics and quantitative genetics, which had long been missing the benefits of advances in sequencing and genomics. Therefore, the application of GS to evaluate its efficiency in yield- and nutrition-related traits in cereals and legumes could be useful. Hyayarimana and Lopez-Cruz [17] used GS to predict the antioxidant production of new and unphenotyped sorghum genotypes. Additionally, the authors tested four GS models, i.e., genomic best linear unbiased prediction, Bayesian ridge regression, Bayesian LASSO, and Bayes B., and reported that based on the accuracies, these GS models can be considered applicable to sorghum breeding for antioxidant production. The second article on the application of GS (optimum index selection) in sorghum demonstrated its suitability for predicting the index selection for multiple but related traits, i.e., dry biomass (above ground), plant height, and dry mass fraction of the fresh material. The utility of GS for these traits can be adapted to predict the performance of future rhizome regrown populations of the same genotypes. Hence, the application of GS as presented in these works could be extended to other cereals for trait improvement [18]. Apart from the application of GS in sorghum, this Special Issue also includes a research article that used a genotyping by sequencing (GBS) approach to dissect the genetic basis of post-flowering drought tolerance in sorghum [19]. Stay-green is an important component of drought-stress tolerance in plants. Previous studies have identified a large QTL interval on SBI-10L. However, owing to its large size and overlap with the root angle, this QTL has limited the gene discovery [20]. Kiranmayee et al. [19] used GBS to fine-map the QTL and genes in a sub-set of 152 F2:3 sorghum progenies and reported seven QTL and single genes that are involved in drought-stress tolerance. This is an important step forward towards breeding drought-tolerant sorghum genotypes using indigenous germplasm.

The advances in molecular markers and genome sequencing have aided the delineation of the genetic basis of agronomic traits [21]. Particularly, the developments in genome sequencing and sequencing technologies have pushed forward the application of genome-wide association studies (GWAS) and QTL mapping and aided in the discovery of important genes related to the ability of a crop plant to withstand biotic and abiotic stresses [22]. However, the application of these techniques in understanding the genetic basis of antioxidants is limited. Nadeem et al. [23] attempted to use GWAS on a panel of common bean landraces and commercial cultivars of Turkish origin. The authors reported four markers (DArTseq marker) with a significant association with the antioxidant activity and suggested the possible role of four putative genes. This study demonstrates that the application of GWAS for complex traits can enable researchers to extract useful information for the genetic improvement of legumes. Another research article included in this Special Issue used marker-assisted backcross breeding for improving stable restorer lines for fungal blast (*Magnaporthe oryzae*) in rice [24]. These articles clearly dictate that the recent developments in genomics can be successfully merged with classical plant breeding to yield crop plants with better potential against biotic and abiotic stresses, and our understanding about the genetic basis of complex pathways can be increased.

*Fusarium solani* f. sp *phaseoli* (FSP) causes Fusarium root rot in common bean and causes up to 84% yield losses [25]. The resistance to FSP is a complex multicomponent system and involves pathogen-associated molecular pattern-triggered immunity as well as effector-triggered immunity. Apart from these, the role of different pathways against root rot such as secondary metabolites, phenylpropanoid, and flavonoid pathways is widely discussed in different crop plants [26,27,28]. This Special Issue contains the first comprehensive transcriptomic and metabolomics response of common bean infected by FSP [14]. This is a major development in understanding the root responses of common bean seedlings after a particular infection time since with the disease progression, the molecular mechanisms of resistance or susceptibility of roots to a particular pathogen could vary [14,29]. It is evident from this study that the developments in genomics have triggered the large-scale response identification. For example, the authors report that FSP induced different pathways in common bean roots for successful invasions. On the other hand, the resistant common bean genotypes responded by modifications in the cell wall, reactive oxygen species, and hormone-driven responses. Hence, such studies open up the interesting roles of different pathways and broaden the possibilities to equip plants with better resistance strategies.

Apart from exploration of FSP resistance in the common bean, this Special Issue also hosts a research paper on heat-stress tolerance in flowering Chinese cabbage (*Brassica campestris* L.) [12]. As discussed in the earlier paragraphs, the major threat to crop production is being posed by the rising global temperatures [2]. Literature published in the last three decades has successfully highlighted the effect of heat stress on crop productivity, including that of Chinese cabbage. MicroRNAs (miRNAs) are small regulatory RNAs that guide gene expression at the post-transcriptional level [30]. The developments in sequencing have enabled large-scale identification of novel and conserved miRNAs that are expressed in a particular condition [31]. Ahmad et al. [12] made an effort to identify novel and reported miRNAs in Chinese cabbage grown under heat stress. The 14 novel miRNAs identified can be important targets for future heat-resistance improvement studies in plants. Heat stress, when combined with drought stress, can cause disproportionate damage to plants and may result in substantial losses in crop yield [32]. Considering this threat to plant productivity and the rising global temperatures and future forecasts (Figure 1b), it is important to identify genes that can endow crop plants with better heat- and drought-stress abilities. To this regard, this Special Issue includes a report on the identification of an expansin-like B1 gene in *Brassica rapa* [33]. The newly identified *BrEXLB1* gene was found to be associated with root development, drought stress, and seed germination. The expansin genes play important roles in cell walls. It is suggested that their extended roles include participation in stress responses, where they interact with internal and external signals to produce a response in plant cells [34]. The authors discovered that the expression of *BrEXLB1* can be triggered by the changes in the endogenous phytohormonal levels in the roots, and infections with clubroot, *Pectobacterium carotovoru,* and turnip mosaic virus [33]. The results on both *Brassica* species provide potential candidate genes and pathways for improving the *Brassica* sp. plants’ responses to biotic and abiotic stresses.

Biotic and abiotic stress tolerance can be managed by different classes of enzymes such as glutathione S-transferases (GSTs) [35]. As we discussed in above paragraph, the genes with a multitude of effects on different pathways can be good candidates for crop improvement. We say this because, in a particular field, a crop plant could be affected by multiple stresses at a time [36]. Thus, engineering plants with genes involved in multiple stress-tolerance pathways could be a wise strategy. To unveil the multifunctionality of these genes, their detailed characterization is required. In this Special Issue, one rice tau class GST (*OsGSTU17*) is examined in detail for its activity and thermal stability [37]. GSTs have high a research value for agricultural production due to their multifunctionality in GSTs conjugate glutathione (GSH) dependent detoxification and GSH-dependent peroxidase activities. Therefore, the study by Yang et al. [37] explores the detailed structure of *OsGSTU17,* and this knowledge can be further used for modifying its activity.

Once the improved cultivars are produced by employing the techniques discussed above, identification and purity assessment are considered an important step in the registration, trading, and selection of suitable cultivars for a target area [38]. DNA-based systems are being deployed for distinctness, uniformity, and stability (DUS) testing across the globe, especially after the recommendation by the International Union for the Protection of New Varieties of Plants [39]. Though this technology seems expansive when it comes to testing a small number of seeds, new cost-effective techniques are being developed, such as reduced representation genome sequencing [40]. One such extensively used technique is specific locus amplified fragment sequencing (SLAF-seq) [41]. In this Special Issue, Zhang et al. [42] use the SLAF-seq-developed single nucleotide polymorphism (SNP) and simple sequence repeat (SSR) markers to test the DUS of five soybean cultivars. The authors report cultivar purity levels of 91.89–93.96% and extend the use of the technique to 150 soybean cultivars of different origin. The results from this study suggest that although the large-scale genome sequencing techniques with higher genome resolution are expansive for such small tasks, there are other techniques being developed that can be employed for such tasks in an economic way [42].

We hope that this Special Issue will provide our readers with a collection of techniques and methods through which the published works have achieved genetic improvement and related objectives. We thank all authors for their contributions and reviewers for their critical assessment of these articles.

## Figures and Tables

**Figure 1:**
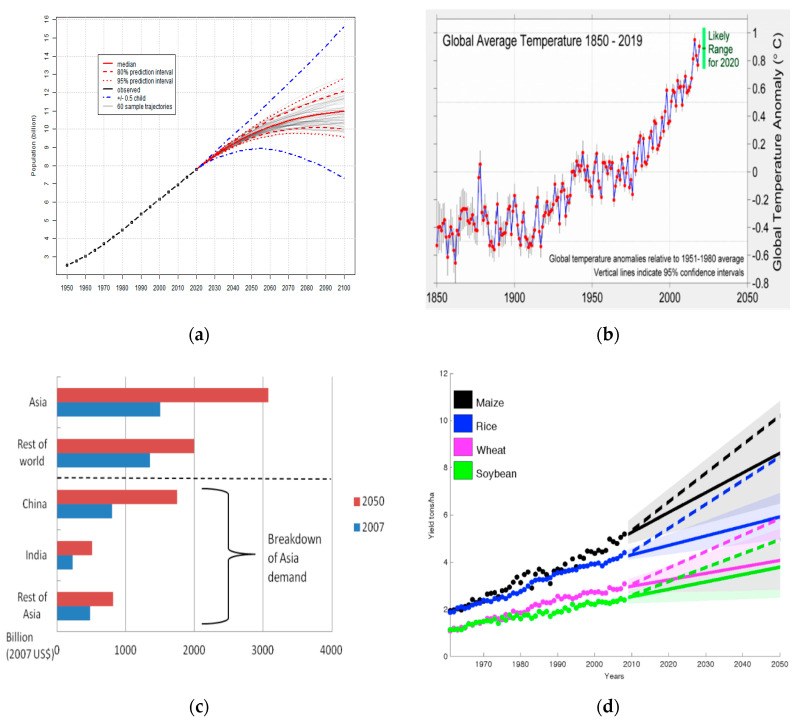
Global trends in (**a**) increase in population (World Population Prospects 2019; http://population.un.org/wpp/), (**b**) average temperature (barkeleyearth.org), (**c**) agrifood demand [4], and (**d**) required yield improvement of four major crops, i.e., maize, rice, wheat, and soybean [5].

**Figure 2:**
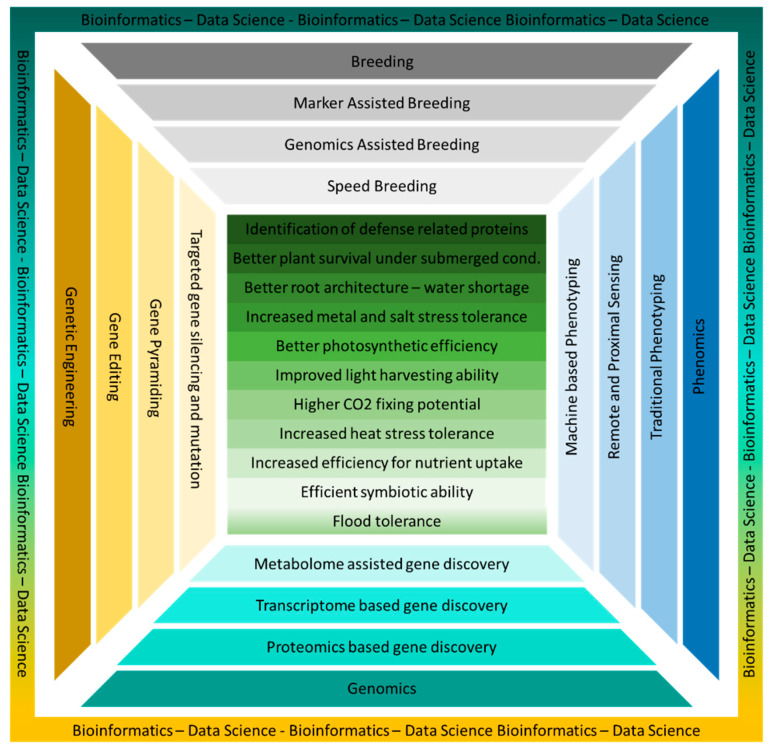
The agronomic traits (given in green boxes) of which improvement can significantly help in achieving higher yield goals in crop plants through the integration of techniques, i.e., plant breeding, genetic engineering, phenomics, and genomics represented by four sides. Bioinformatics and data science are two emerging disciplines that are helping to elaborate the large-scale plant genome/proteome/transcriptome/metabolome data in a meaningful way.
